# The Effect of Probiotics (MCP^®^ BCMC^®^ Strains) on Hepatic Steatosis, Small Intestinal Mucosal Immune Function, and Intestinal Barrier in Patients with Non-Alcoholic Fatty Liver Disease

**DOI:** 10.3390/nu13093192

**Published:** 2021-09-14

**Authors:** Mohamad Hizami Mohamad Nor, Nurainina Ayob, Norfilza M. Mokhtar, Raja Affendi Raja Ali, Geok Chin Tan, Zhiqin Wong, Nor Hamizah Shafiee, Yin Ping Wong, Muaatamarulain Mustangin, Khairul Najmi Muhammad Nawawi

**Affiliations:** 1 Gastroenterology and Hepatology Unit, Department of Medicine, Faculty of Medicine, Universiti Kebangsaan Malaysia, Kuala Lumpur 56000, Malaysia; hizami84@gmail.com (M.H.M.N.); draffendi@ppukm.ukm.edu.my (R.A.R.A.); wzhiqin@ppukm.ukm.edu.my (Z.W.); 2 Department of Physiology, Faculty of Medicine, Universiti Kebangsaan Malaysia, Kuala Lumpur 56000, Malaysia; ainina.ayob@yahoo.com (N.A.); norfilza@ppukm.ukm.edu.my (N.M.M.); 3 GUT Research Group, Faculty of Medicine, Universiti Kebangsaan Malaysia, Kuala Lumpur 56000, Malaysia; tangc@ppukm.ukm.edu.my; 4 Department of Pathology, Faculty of Medicine, Universiti Kebangsaan Malaysia, Kuala Lumpur 56000, Malaysia; ypwong@ppukm.ukm.edu.my (Y.P.W.); amar@ppukm.ukm.edu.my (M.M.); 5 Dietetics Programme, Faculty of Health Sciences, Universiti Kebangsaan Malaysia, Kuala Lumpur 56000, Malaysia; mizahnur91@gmail.com

**Keywords:** NAFLD, probiotics, gut microbiota, mucosal immune function, intestinal permeability

## Abstract

Treatment for non-alcoholic fatty liver disease (NAFLD) currently consists of lifestyle modifications such as a low-fat diet, weight loss, and exercise. The gut microbiota forms part of the gut–liver axis and serves as a potential target for NAFLD treatment. We investigated the effect of probiotics on hepatic steatosis, fibrosis, and biochemical blood tests in patients with NAFLD. At the small intestinal mucosal level, we examined the effect of probiotics on the expression of CD4+ and CD8+ T lymphocytes, as well as the tight junction protein zona occluden-1 (ZO-1). This was a randomized, double-blind, placebo-controlled trial involving ultrasound-diagnosed NAFLD patients (*n* = 39) who were supplemented with either a probiotics sachet (MCP^®^ BCMC^®^ strains) or a placebo for a total of 6 months. Multi-strain probiotics (MCP^®^ BCMC^®^ strains) containing six different *Lactobacillus* and *Bifidobacterium* species at a concentration of 30 billion CFU were used. There were no significant changes at the end of the study in terms of hepatic steatosis (probiotics: −21.70 ± 42.6 dB/m, *p* = 0.052 vs. placebo: −10.72 ± 46.6 dB/m, *p* = 0.29) and fibrosis levels (probiotics: −0.25 ± 1.77 kPa, *p* = 0.55 vs. placebo: −0.62 ± 2.37 kPa, *p* = 0.23) as measured by transient elastography. Likewise, no significant changes were found for both groups for the following parameters: LiverFAST analysis (steatosis, fibrosis and inflammation scores), alanine aminotransferase, total cholesterol, triglycerides, and fasting glucose. In the immunohistochemistry (IHC) analysis, no significant expression changes were seen for CD4+ T lymphocytes in either group (probiotics: −0.33 ± 1.67, *p* = 0.35 vs. placebo: 0.35 ± 3.25, *p* = 0.63). However, significant reductions in the expression of CD8+ T lymphocytes (−7.0 ± 13.73, *p* = 0.04) and ZO-1 (Z-score = −2.86, *p* = 0.04) were found in the placebo group, but no significant changes in the probiotics group. In this pilot study, the use of probiotics did not result in any significant clinical improvement in NAFLD patients. However, at the microenvironment level (i.e., the small intestinal mucosa), probiotics seemed to be able to stabilize the mucosal immune function and to protect NAFLD patients against increased intestinal permeability. Therefore, probiotics might have a complementary role in treating NAFLD. Further studies with larger sample sizes, a longer duration, and different probiotic strains are needed to evaluate the real benefit of probiotics in NAFLD.

## 1. Introduction

Non-alcoholic fatty liver disease (NAFLD) refers to the presence of hepatic steatosis in the absence of other causes of heavy hepatic fat accumulation, such as heavy alcohol intake. It is one of the most common causes of chronic liver disease nowadays. It has an estimated global prevalence of 25%, with the highest prevalence in the Middle East and South America (31.8% and 30.5% respectively) [1]. A recent meta-analysis on the prevalence of NAFLD in Asian countries (*n* = 237 studies with 13,044,518 individuals as pooled participants), revealed an overall NALFD prevalence of 29.6%, with an increasing prevalence trend over time (1999–2005: 25.3%; 2006–2011: 28.5%; 2012–2017: 33.9%) [2]. This has given rise to a new epidemic in chronic liver disease and increased disease burden.

Treatment options for the NAFLD are limited and mainly revolve around lifestyle interventions such as weight loss via dietary therapy and exercise [3]. It has to be treated early owing to its tendency to progress to end-stage liver cirrhosis and the possible subsequent complication of hepatocellular carcinoma. As our understanding of the pathogenesis of NAFLD has evolved, it has been suggested that disturbances in the gut microbiota composition, leading to gut dysbiosis that can lead to gut–liver axis derangement, is one of the possible factors that triggers local inflammatory cascades [4,5]. Gut dysbiosis theoretically disrupts the arrangement of the adjacent intestinal epithelial cells by loosening tight junctions, which subsequently triggers the response of adaptive immunity. Tight junction proteins such as zona occludens-1 (ZO-1), which is considered to be one of the more important components in junctional complexes, plays a significant role in maintaining the monolayer integrity of epithelial cells through cell–cell communication [6]. The translocation of microbial endotoxins, such as lipopolysaccharides, have been shown to induce steatosis, inflammation, and fibrosis, as well as elevated inflammatory cytokines, such as TNF-alpha (TNF-α) [7]. Therefore, probiotics present a possible target of treatment by manipulating the gut microbiota and modulating intestinal permeability and local mucosal inflammation.

Several methods are available for manipulating the microbiota and its influence on the gut–liver axis, such as the use of prebiotics, probiotics, synbiotics, or faecal microbiota transplants. Probiotics are live microorganisms that, when administered in adequate amounts, confer a health benefit on the host [8]. Prebiotics are selectively indigestible fermented compounds that can induce the growth or activity of beneficial microorganisms [9]. Synbiotics, on the other hand, are a combination of both prebiotics and probiotics. A number of studies involving animals and humans have demonstrated the benefits of pro/synbiotics in NAFLD, such as improving the hepatic steatosis level, reducing hepatic inflammation, and improving biochemical parameters such as alanine aminotransferase (ALT), fasting glucose, and lipid profiles [10,11,12].

In other diseases, gut microbiota manipulation has been shown to improve outcomes. In an animal study, the use of resistant starch to alter the gut microbiota by shifting the composition of the gut microbiota towards butyrate-producing bacteria, was shown to slow the progress of chronic kidney disease in a mouse model of 5/6 nephrectomy [13]. The use of *L. mucosae A1* has been shown to reduce severe lipid accumulation in the serum, liver, and aortic sinus of ApoE-/-mice on a Western diet, while also reducing the serum lipopolysaccharide-binding protein content of mice, reflecting improved metabolic endotoxemia [14]. In humans, the use of *Lactobacillus rhamnosus GG-*supplemented formula was shown to increase tolerance of infants to cow’s milk allergy by expanding the butyrate-producing bacterial strains in the gut [15]. A larger meta-analysis of supplementation with the same strain was shown to reduce antibiotic-associated diarrhea for any reason [16]. Furthermore, 6 months of supplementation with probiotics (MCP^®^ BCMC^®^ strains) containing six viable microorganisms of *Lactobacillus* and *Bifidobacterium* strains in post-surgical colorectal cancer patients was shown to reduce the level of pro-inflammatory cytokines (TNF-α, IL-6, IL10, IL-12, IL-17A, IL-17C, and IL-22) compared with the pre-treatment level [17].

Hence, we conducted a randomized, double-blind, placebo-controlled trial to assess whether probiotic supplementation can improve hepatic steatosis, fibrosis, and other clinical biomarkers in NAFLD patients. Since the small intestine is responsible for most nutrient absorption and digestion, it is prudent to explore the role of the small intestine in the development of NAFLD. Therefore, we also determined the effect of probiotics on changes in the expression of CD4+ and CD8+ T lymphocytes (mucosal immune function) as well as ZO-1 (small intestinal barrier).

## 2. Methodology

### 2.1. Study Design

A randomized, double-blind, placebo-controlled pilot study involving patients from the Universiti Kebangsaan Malaysia Medical Centre (UKMMC) was conducted, and the protocol was approved by the institutional ethics committee (UKM PPI/111/8/JEP-2019-456). The trial was registered at the US National Institutes of Health website (http://www.clinical-trials.gov) #NCT04074889.

### 2.2. Patient Recruitment

The inclusion criteria were: patients aged 18 years old and above with an ultrasound diagnosis of fatty liver, a baseline-controlled attenuation parameter (CAP) score measured by FibroScan of ≥263 dB/m, and a baseline ALT of more than 35 IU/L for males and 25 IU/L for females. Patients with evidence of other chronic liver diseases, such as concomitant hepatitis B or C infections, and autoimmune hepatitis disorder or alcoholic liver disease, were excluded from this study. Other exclusion criteria consisted of evidence of acute disorders affecting the liver such as drug-induced liver injury, the presence of hepatocellular carcinoma (or liver metastases), any biliary diseases (which would explain the raised ALT, such as gallstones), or evidence of liver cirrhosis. Patients were advised to stop taking any nutritional supplements and to temporarily discontinue any lipid-lowering drugs, beginning at least 4 weeks prior to the study. The recruitment period lasted for a period of 6 months (September 2019 to February 2020).

### 2.3. Clinical Assessment and Intervention

At baseline measurement, patients’ comorbidities (diabetes mellitus, hypertension and dyslipidemia) were recorded. Body mass index (BMI) classifications were performed as per Malaysian clinical practice guidelines [18]. Therefore, underweight refers to a BMI of <18.5 kg/m^2^, normal weight a BMI of 18.5–22.9 kg/m^2^, pre-obesity a BMI of 23.0–27.4 kg/m^2^, and obesity a BMI of >27.5 kg/m^2^. Blood samples were investigated and a transient elastography was also performed. These tests will be elaborated in the next section.

The baseline dietary pattern was assessed using a Food Frequency Questionnaire (FFQ), a semiquantitative tool for assessing the dietary patterns of patients (adjusted for the Malaysian diet) at the baseline and after the intervention [19]. The FFQ was analyzed to ensure there were no significant differences in the nutritional intake between the two groups, before and after the intervention. All patients were instructed to maintain their current diet and lifestyles. The patients were also instructed to not embark on any weight loss or diet program.

The random allocation sequence was generated by a computer model using Microsoft Excel to create blocks of four in order to allow even numbers in each interventional arm. The randomization was performed by the principal investigators, who held the allocation sequence. After the baseline measurement had been completed, sachets containing the probiotics or a placebo were given to the participants according to their assigned group. Both the investigators and the patients were blinded to the content of the sachets, and an independent investigator held the code, which was revealed at the end of the study.

### 2.4. Laboratory Investigations

A set of blood investigations was carried out before and after the intervention that included triglycerides (TG), alpha-2-macroglobulin, total cholesterol (TC), gamma-glutamyl transferase (GGT), ALT, aspartate transaminase (AST), total bilirubin, fasting glucose, haptoglobin, and apolipoprotein A1. These measurements were then inputted into an algorithm developed by Fibronostics (LiverFAST) to estimate the level of steatosis, fibrosis, and inflammation based on the blood results. This algorithm has been shown to have a prediction outcome in terms of steatosis and fibrosis that is comparable with liver stiffness measurements measured by transient elastography and liver biopsy, as it can objectively estimate the level of steatosis, fibrosis, and inflammation to be not significant, minimal, moderate, significant, or severe [20,21].

### 2.5. Immunohistochemistry Analysis

Duodenal samples (the second part of the duodenum) from the NAFLD patients were obtained by performing oesophagogastroduodenoscopy (OGD) before and after the intervention. Immunohistochemistry was performed on serial 3 mm sections of the formalin-fixed paraffin-embedded duodenum biopsy samples. The slides were treated with rabbit monoclonal CD4 antibody (Cell Marque, Sigma Aldrich, Burlington, VT, USA) at a dilution of 1:100, mouse monoclonal CD8 antibody (Dako, Agilent Technologies, Santa Clara, CA, US), and rabbit monoclonal to ZO-1 tight junction antibody (Abcam, Cambridge, UK) at a dilution of 1:300. The slides were incubated for 30 min at room temperature, followed by treatment with secondary antibodies and horseradish peroxidase (HRP) for another 30 min. The slides were finally visualized with diaminobenzidine, counterstained with hematoxylin, dehydrated, and mounted. Brown staining of CD4+, CD8+ and ZO-1 on the cell cytoplasm and membrane was classified as positive staining. External positive controls were always included in the batch of slides (CD4+ and CD8+: tonsil; ZO-1: kidney).

The immunostaining was reviewed and scored independently by two histopathologists. We counted the average number of CD4+ or CD8+ T lymphocytes in at least three selected villi, which was later analyzed as the mean number of labelled nuclei over a total of 100 enterocytes. In the lamina propria, the staining was examined semi-quantitatively in the cytoplasmic area and scored as follows: 0: no staining; +: focal staining; ++: regional staining; and +++: no loss. For ZO-1, the results were expressed semi-quantitatively, as previously reported [22]; in brief, cytoplasmic labelling intensity was scored as follows: 0: complete loss; +: moderate loss; ++: focal loss; +++: very focal loss; and ++++: no loss. The staining index score for CD4+, CD8+, and ZO-1 was based on the immunopositive area.

### 2.6. Transient Elastography

A FibroScan 502, manufactured by Echosens (Paris, France) was used in the study to obtain the liver stiffness measurement (LSM, kPa) and the controlled attenuation parameter (CAP, dB/m). A CAP score of ≥263 was taken as the cut-off value to indicate the presence of hepatic steatosis [23]. The measurement was considered reliable if there were at least 10 valid readings, a success reading rate of at least 70%, and an interquartile range/median (IQR/M) of less than 20%. The same equipment was used throughout the study, and all the measurements were performed only by the trained principal investigator of this study in order to eliminate inter-operator variability.

### 2.7. Probiotics and Compliance

The probiotics used were Microbial Cell Preparation (MCP) (Hexbio^®^; comprising MCP^®^ BCMC^®^ strains) from B-Crobes Laboratory Sdn. Bhd. Their specifications are listed in Table 1. Patients were instructed to consume the product twice a day (in the morning and evening, either with or without meals). The product can be mixed with one glass of water (approximately 250 mL) before consumption, or consumed directly. On the other hand, the placebo contained the same excipients but without the live bacteria. The content of both the probiotics and placebo were packed in a similar-looking sachet and were indistinguishable from each other both in terms of colour, taste, and smell, and were labelled as A or B. Sachets were kept in a dry place below 25 °C and away from direct sunlight. The subjects of the study were also told to do the same. The shelf life of the sachets was 2 years, and the sachets were delivered to the study site at 3-month intervals.

Compliance was checked via the sachet count method during the 3-month appointment with the subjects and another count at the end of the study. A periodic check was also carried out via phone calls and text messages. We accepted compliance rates between 85% and 100% [24].

### 2.8. Statistical Analysis

Descriptive statistics were computed and are presented as means ± standard deviation, as the data were normally distributed. All statistical analyses were performed using SPSS software version 20.0 (SPSS Inc. Chicago, IL, USA) for Windows. Demographic data are presented as means and standard deviations for the normally distributed data. The data were analyzed with a paired sample t-test to account for reductions within the group (probiotics or placebo). An independent sample t-test was used to compare the mean reduction between the groups. For non-normally distributed data, the values are presented as medians (IQR) and the mean differences were calculated using Wilcoxon signed-rank tests. For the IHC analysis, paired sample t-tests were performed to determine changes in the number of CD4+ and CD8+ T lymphocytes in the villi, while the Wilcoxon signed-rank test was used for cytoplasmic staining of CD4+, CD8+, and ZO-1 in the lamina propria, villi, and crypts. A *p*-value of ≤0.05 was considered to be statistically significant.

### 2.9. Primary and Secondary Outcomes

The primary outcome was the mean difference in hepatic steatosis, as measured by CAP in dB/m by FibroScan after probiotics/placebo supplementation. Secondary outcomes included the mean difference in fibrosis score (as measured by FibroScan), the mean difference in selected liver enzymes and lipid profiles (which includes serum total cholesterol, triglycerides, ALT, AST, GGT and fasting blood glucose), and the computed scores (LiverFAST), such as the steatosis, fibrosis, and activity (correlating to hepatic inflammation) scores. The other secondary outcomes were the mean differences in CD4+ and CD8+ T lymphocyte counts observed in the villi, the percentage of proteins in areas of the lamina propria, as well as in the villi and crypts for the tight junction protein ZO-1.

## 3. Results

### 3.1. Baseline Characteristics

**Figure 1:**
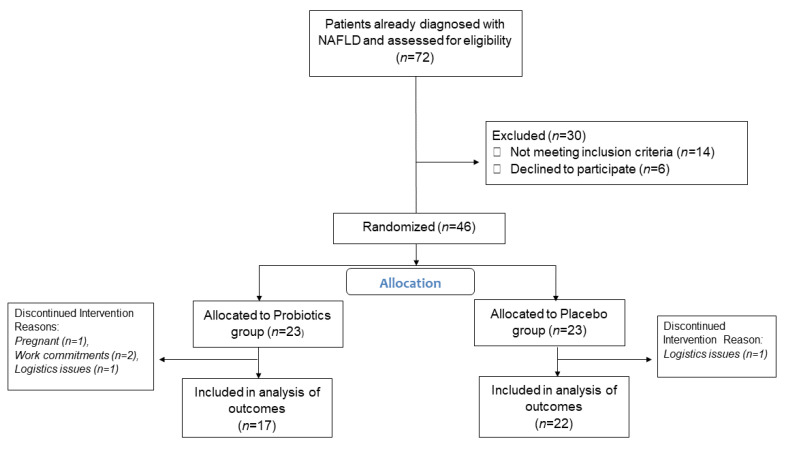
Consolidated Standards of Reporting Trials flow diagram of the study participants. NAFLD, non-alcoholic fatty liver disease.

Eighty-five percent of the total patients enrolled in the study completed the intervention, with 15 patients in the probiotics group and 17 patients in the placebo group, as shown in Figure 1. The baseline demographics data are summarized in Table 2. The reasons for dropout included an inability to come to the follow-up appointments due to work commitments, getting pregnant during the trial, and logistics issues of the patients. None of the studied patients had any compliance issues related to intolerance or side-effects of the probiotics/placebo. The majority of the participants were in the obese category. No statistically significant difference existed between the two groups regarding their baseline anthropometric measurements, transient elastography, or biochemical blood tests. Data were analyzed per-protocol.

### 3.2. Hepatic Steatosis, Fibrosis and Activity Scores

The post-intervention hepatic steatosis scores in the probiotics group showed a greater reduction in the mean CAP score, from 339.17 ± 33.58 dB/m to 317.41 ± 40.37 dB/m (a mean reduction of −21.70 dB/m, *p* = 0.052) compared with that of the placebo group, which was from 329.18 ± 35.15 dB/m to 318.45 ± 45.37 dB/m (a mean reduction of −10.72 dB/m; *p* = 0.29). However, the improvement in CAP scores in the probiotics group was not statistically significant. For the hepatic fibrosis score measured using FibroScan, there was no statistically significant difference within the probiotics (from 7.25 ± 2.76 kPa to 6.99 ± 2.74 kPa; *p* = 0.55) and placebo (from 7.58 ± 2.82 kPa to 6.95 ± 2.19 kPa; *p* = 0.23) groups.

LiverFAST was also used to evaluate the hepatic steatosis, fibrosis, and inflammatory activity scores in NAFLD patients. For the probiotics group after supplementation with probiotics, we did not elicit any improvement in the scores for all three parameters (steatosis score: *p* = 0.06; fibrosis score: *p* = 0.88; activity score: *p* = 0.78). Likewise, no improvement in the score was seen in the placebo group (steatosis score: *p* = 0.053; activity score: *p* = 0.57). In fact, the median fibrosis score showed a significant increase after the 6-month study period from 0.27 (0.15–0.45) to 0.33 (0.15–0.36) (*p* = 0.022; Table 3 and Table 4).

### 3.3. Biochemical Blood Tests

Other biochemical parameters such as ALT, AST, GGT, total cholesterol, triglycerides, and fasting glucose did not show any significant differences within both groups after the intervention (Table 3 and Table 4).

### 3.4. Immunohistochemistry Analysis

#### 3.4.1. Expression of CD4+ T Lymphocytes

**Figure 2:**
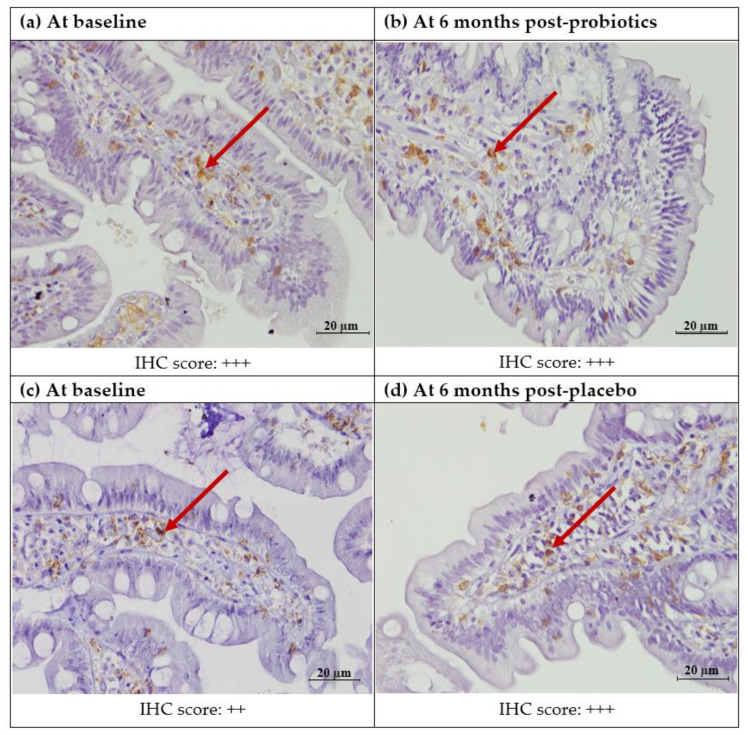
Immunohistochemicalstaining of CD4+ protein in the duodenal mucosa (CD4+, 400X). (**a**) Duodenal mucosa of a patient with NAFLD at baseline. (**b**) Duodenal mucosa of a patient with NAFLD after 6 months of probiotics. (**c**) Duodenal mucosa of a patient with NAFLD at baseline. (**d**) Duodenal mucosa of a patient with NAFLD after 6 months of the placebo. The arrows show brownish staining of the CD4+ T lymphocytes in the lamina propria. Semi-quantitatively, both the probiotics and placebo groups did not show any difference in the percentage of staining before and after the intervention. Staining score: 0: no staining; +: focal staining; ++: regional staining; and +++: no loss.

**Figure 3:**
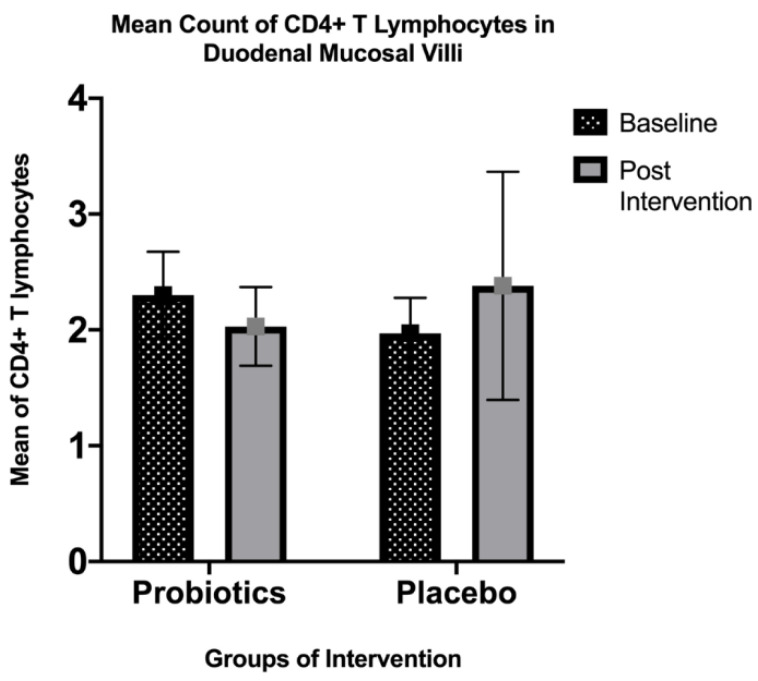
Immunohistochemical staining analysis of CD4+ T lymphocytes in the villi of the duodenal mucosa in NAFLD patients. There was a slight decrease in the mean count of intraepithelial CD4+ T lymphocytes observed in the probiotics group after 6 months of the intervention (from 2.30 ± 1.83 to 1.97 ± 1.50; *p* = 0.35), while the placebo group showed a slight increase in the mean intraepithelial CD4+ T lymphocyte count (from 2.03 ± 1.68 to 2.38 ± 4.82; *p* = 0.63). Both groups did not show any significant changes.

Quantitatively, there was no significant difference in the mean post-intervention CD4+ T lymphocyte count in the small intestinal villi for both the probiotics (from 2.30 ± 1.83 to 1.97 ± 1.50; *p* = 0.35) and placebo (from 2.03 ± 1.68 to 2.38 ± 4.82; *p* = 0.63) groups. Similarly, the cytoplasmic staining of the small intestinal lamina propria did not show any significant change in expression for both groups (probiotics: Z-score = 0.00, *p* = 1.00; placebo: Z-score= −0.302, *p* = 0.76) (Figure 2 and Figure 3).

#### 3.4.2. Expression of CD8+ T Lymphocytes

**Figure 4:**
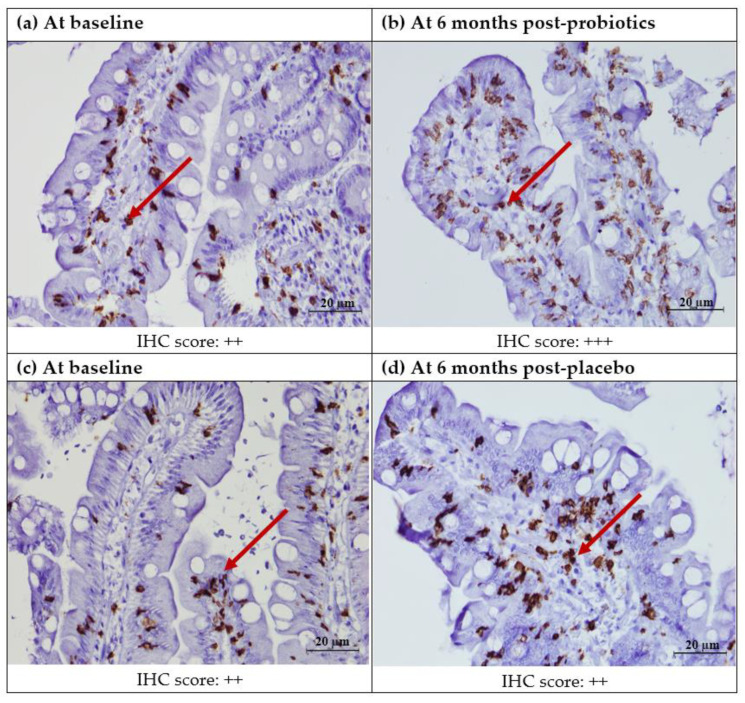
Immunohistochemical staining of CD8+ protein in the duodenal mucosa (CD8+,400X). (**a**) Duodenal mucosa of a patient with NAFLD at baseline. (**b**) Duodenal mucosa of a patient with NAFLD after 6 months of probiotics. (**c**) Duodenal mucosa of a patient with NAFLD at baseline. (**d**) Duodenal mucosa of a patient with NAFLD after 6 months of the placebo. Red arrows show brownish CD8+ T lymphocytes in the lamina propria. Semi-quantitatively, no significant post-intervention difference was seen in either the probiotics of placebo groups. Staining score: 0: no staining; +: focal staining; ++: regional staining; and +++: no loss.

**Figure 5:**
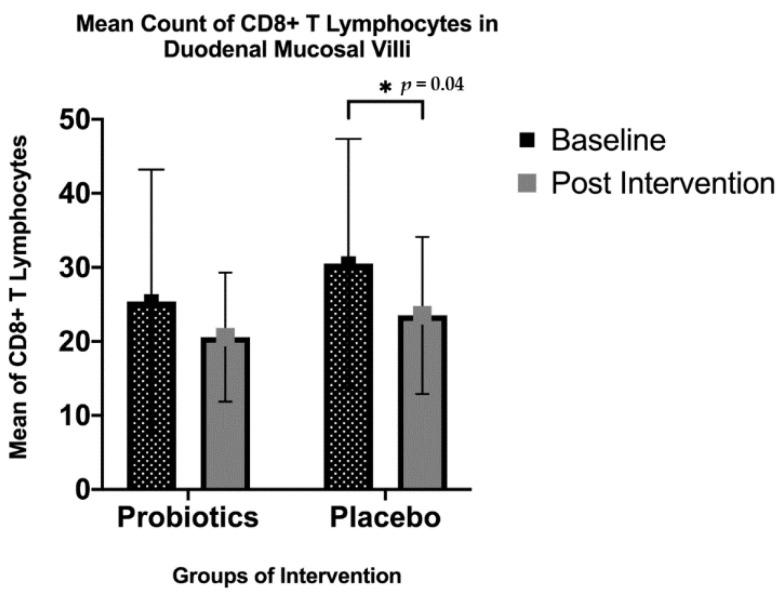
Immunohistochemical staining analysis of CD8+ T lymphocytes in the villi of the duodenal mucosa in NAFLD patients. There was a significant decrease in the mean count of intraepithelial CD8+T lymphocytes observed in the placebo group after 6 months of the intervention (from 30.51 ± 16.85 to 23.51 ± 10.61, * *p* = 0.04), while the probiotics group showed a slight decrease in the mean intraepithelial CD8+T lymphocyte count; however, this was not statistically significant (*p* = 0.211).

The quantitative analysis in the small intestinal villi showed that there was a significant decrease in the CD8+ T lymphocyte count for the placebo group (from 30.51 ± 16.85 to 23.51 ± 10.61; *p* = 0.04), but not for the probiotics group (from 25.40 ± 17.81 to 20.58 ± 8.72; *p* = 0.21). However, the same trend was not seen in the semi-quantitative analysis for cytoplasmic staining in the lamina propria (Figure 4 and Figure 5).

#### 3.4.3. Expression of ZO-1

**Figure 6:**
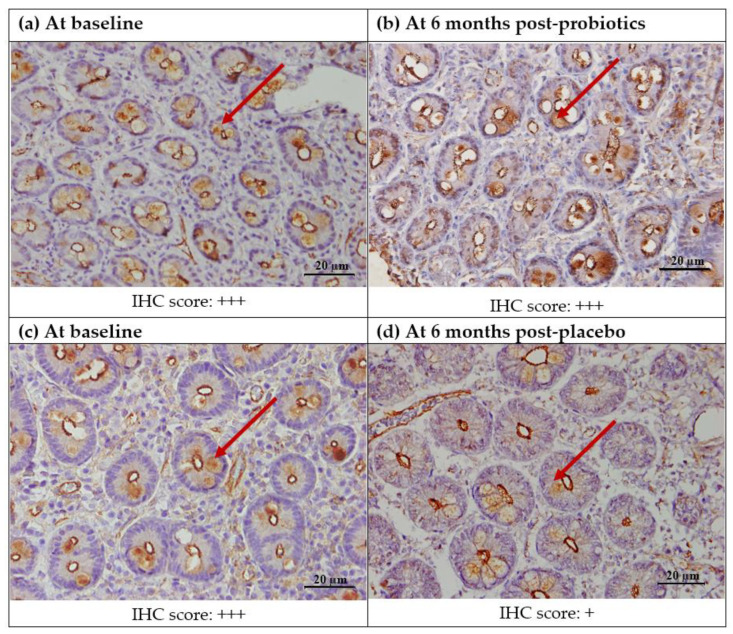
Immunohistochemical staining of tight junction zonula occluden-1 (ZO-1) protein in the duodenal mucosa (ZO-1400X). (**a**) Duodenal mucosa of a patient with NAFLD at baseline. (**b**) Duodenal mucosa of a patient with NAFLD after 6 months of probiotics. (**c**) Duodenal mucosa of a patient with NAFLD at baseline. (**d**) Duodenal mucosa of a patient with NAFLD after 6 months of the placebo. Brownish ZO-1 IHC staining was observed in the intestinal crypts (red arrows). There was a significant reduction in ZO-1 expression in the post-intervention placebo group (Z-score = −2.86, *p* = 0.04) but not in the probiotics group (Z-score = −0.93, *p* = 0.35). Staining score: 0: complete loss; +: moderate loss; ++: focal loss; +++: very focal loss; and ++++: no loss.

There was no significant change in ZO-1 expression after the intervention for both groups, when analyzed semi-quantitatively in the small intestinal villi (probiotics: Z-score = −0.97, *p* = 0.33; placebo: Z-score = −0.73, *p* = 0.47). On the other hand, semi-quantitative analysis in the crypt area showed a significant reduction in ZO-1 expression for the placebo group (Z-score = −2.86, *p* = 0.04) but no significant change for the probiotics group (Z-score = −0.93, *p* = 0.35) (Figure 6).

### 3.5. Nutritional Analysis

Based on the FFQ, there was a significant decrease in the daily total fat intake in the probiotics group after the intervention compared with the placebo group (−10.63 ± 18.56 g, *p* < 0.05 vs. −2.53 ± 21.32 g, *p* = 0.58). Other macronutrients showed no significant difference in either of the two groups, as shown in Table 5.

LiverFAST categories:

(I) Fibrosis score: 0–0.27, no fibrosis, F0; 0.28–0.48, minimal, F1; 0.49–0.58, moderate, F2; 0.59–0.74, significant, F3; 0.75–1.00, severe, F4
(II) Steatosis score: 0.69–1.0, no steatosis, S0; 0.38–0.56, minimal, S1; 0.57–0.68, moderate, S2; 0.69–1.00, severe, S3
(III) Activity score: 0–0.29, no activity, A0; 0.3–0.52, minimal, A1; 0.53–0.62, moderate, A2; 0.63–0.72, significant, A3; 0.73–1.00, severe, A4

## 4. Discussion

There was no specific gut microbiota associated with NAFLD. However, multiple studies have shown how a dysbiotic environment exists in NAFLD and non-alcoholic steatohepatitis (NASH) patients. Michail et al. (2015) compared three groups, which were lean healthy children, obese children without NAFLD, and obese children with NAFLD. Using faecal samples, they found that children with NAFLD had more *Gammaproteobacteria* and *Epsilonproteobacteria* (at class level) and *Prevotella* (at genus level) compared with healthy controls [25]. Zhu et al. (2012) found a higher prevalence of *Proteobacteria* (phylum level), *Enterobacteriaceace* (family level), and *Escherichia* (genus level) in children with NASH [26]. In contrast to the study by Zhu et al., Mouzaki et al. (2013) found a lower percentage of *Bacteroidetes* in NASH patients compared with healthy controls, independent of their diet or body mass index [27]. On the other hand, Wong et al. (2015) did not find any change in *Bacteroidetes* levels between the NASH patients and healthy controls [28]. Instead, the abundance of *Firmicutes* decreased in the NASH patients compared with healthy controls. These inconsistencies may be due to various factors, such as the different geographical locations, diets, ages, and the population studied [29].

There have been multiple studies concerning the manipulation of the gut microbiota in order to achieve a clinical improvement in hepatic steatosis and inflammation, together with other measured laboratory parameters, using either prebiotics, probiotics or synbiotics. Mofidi et al. (2017), in a randomized placebo-controlled trial involving 50 patients, were able to demonstrate a greater reduction in hepatic steatosis and fibrosis measured by transient elastography. The researchers used synbiotics consisting of multiple strains of *Lactobacillus* sp. and *Bifidobacterium* sp. compared with a placebo group over 28 weeks [10]. Similarly, Eslamparast et al. (2014), in a study involving 52 patients, were also able to demonstrate a greater reduction in ALT, GGT, and high sensitivity C-reactive protein in the synbiotics group compared with the placebo group [30]. A meta-analysis in 2013 of four other randomized controlled trials also showed the positive effect of probiotics in reducing ALT and total cholesterol [31]. Duseja et al. (2019) demonstrated an improvement in the liver histology of patients with NAFLD after taking probiotics compared with the usual care group [32]. However, most recently in 2020, Scorletti et al., in one of the largest studies, which involved 104 patients for a duration of 10 to 14 months, revealed no significant difference in liver steatosis in both the synbiotics and placebo groups [33].

Probiotics have been shown to affect the mucosal immune function, as well as the intestinal barrier in fatty liver subjects or models. Jiang et al. (2015) revealed a decreased number of duodenal CD4+ and CD8+ T lymphocytes in a NAFLD group compared with healthy controls [34]. This finding was in agreement with other recent studies by Zhao et al. (2021) and Ma et al. (2016), which also found a selective loss of intrahepatic CD4+ T lymphocytes in both human and animal models due to lipid metabolism dysregulation [35,36]. Another new published study by Antonucci et al. (2020) showed the suppression of circulating CD4+ and CD8+ T lymphocytes activation due to infiltration of polymorphonuclear neutrophils (PMNs), which were elevated in NAFLD and NASH patients [37]. However, a few studies that used liver tissues instead of duodenal tissues displayed contrasting results. A study by Her et al. (2020) conducted on a humanized mouse model with an induced high-fat high-calorie diet (HFHD), which showed an increasing trend of effector memory T cells and human CD4+ after 20 weeks of observation [38]. This can also be seen in another study by Hu et al. (2016), which concluded that a high activation of hepatic CD4+ and CD8+ T lymphocytes was due to gut-derived lymphocytes that migrated to the liver [39]. The study indicated that immune cells with different localizations might exhibit different immune responses.

Zonula occluden proteins, which comprise ZO-1, ZO-2, and ZO-3, are tight junction proteins that are responsible for controlling the paracellular pathway of solutes between the linings of adjacent epithelial cells [40]. Miele et al. (2009) found a disruption of intestinal tight junction ZO-1 with evidence of a small increase in intestinal bacteria overgrowth in biopsy-proven NAFLD patients [22]. An animal study by Feng et al. (2019) manipulated ApoE^−/+^ mice with a standard and high-fat diet (HFD) supplemented with curcumin for 16 weeks [41]. The expression of ZO-1 was upregulated in HFD mice supplemented with curcumin, in concordance with reduced circulating lipopolysaccharide levels. An animal study by Kim et al. (2019) reported upregulation of ZO-1, Claudin-3, and Mucin-4 in a dextran sodium sulphate-induced model after *Lactobacillus paracasei* treatment for 8 days [42].

In our study, we did not find a statistically significant reduction in the level of hepatic steatosis for the probiotics group. Additionally, no improvement was seen in other biochemical blood parameters. To our knowledge, our study is currently the first to be conducted in the local Malaysian setting. Malaysia, compared with other countries, has a more diversified dietary intake, due to the multiethnic population in the country. So far, data on gut microbiota prevalence in Malaysia are still limited. Chong et al. (2015) revealed that the northern Malaysian population has high levels of *Bacteroidetes* and *Firmicutes* (phyla). In their sub-analysis of Aboriginal children, an abundant amount of *Aeromonadales* (order) was seen in comparison with the more dominant presence of *Bacteroidetes* and *Firmicutes* [43]. Neoh et al. (2018), in a study of 15 patients living in urban areas, showed three prevalent gut phyla: *Bacteroidetes, Firmicutes,* and *Fusobacterium* [44]. In addition, Lee et al. (2014), in a sub-analysis, revealed how the Malaysian gut microbiota was much more diverse compared with that of people who live in New York [45]. The differing local gut microbiota may have affected the results of this study. Further data on the local gut microbiota may be needed, and a larger sample size may be suggested if similar studies are repeated in a local population at a later time.

Our study’s findings are comparable with some previous studies, in which the placebo group showed a significant reduction in expression of CD8+ T lymphocytes and ZO-1 after 6 months. Although we failed to demonstrate a significant improvement in the expression of CD8+ T lymphocytes and ZO-1 for the probiotics group, we revealed no significant reduction in their expression, unlike in the placebo group. Therefore, this study gives an insight into the thought that probiotics might play a role in stabilizing mucosal immune function, as well as preventing intestinal permeability in NAFLD patients.

There are several limitations of the study that need to be mentioned. The sample size for the study was relatively small and may have led to a high probability of Type I statistical errors and a higher chance of confounding factors affecting the results. Participants were also instructed not to significantly change their diet during the study, but we found a significant decrease in total fat intake in the probiotics group, which may also have affected the final results. However, the anorexigenic effect of probiotics has been studied in several animal models and human studies; for example, Bjerg et al. (2014) showed that intraluminal infusion of *Lactobacillus casei W8* into an ex vivo porcine ileum resulted in increased *GLP-1* secretion, which can potentially suppress the appetite acutely. Ingestion of a high concentration of *Lactobacillus casei W8* prior to ad libitum lunch also resulted in a lower energy intake compared with those who consumed the placebo [46]. Although the fat intake was reduced in our probiotics group, it is worth mentioning that it still remains within the range recommended by the Malaysian nutritional committee and the joint FAO/WHO recommendations [47,48] However, our baseline characteristics indicated a diet rich in protein, as the total intake exceeded the recommended nutritional guidelines (61 g/day for men and 52 g/day for women). The total fat intake seems to be within the recommended intake (61 to 73 g for men and 53 to 63 g for women). The carbohydrate intake was also within the recommended level, not exceeding 50% of the daily total energy intake. This trend of higher dietary protein intake compared with carbohydrates and other macronutrients has been shown in several studies looking at the average dietary trends of various cohorts of the Malaysian population, either in central urban populations or in suburban East Malaysian populations [49,50]. This trend, however, has been observed globally and is predominant in countries with emerging economies where the improving economic status enables its population to consume more foods of animal origin; hence, we did not expect significant changes in our population’s diet compared with many developing and developed countries [51]. Although FibroScan has been proven to have a good correlation with liver histology in NAFLD, liver biopsy still remains the gold standard of liver histology assessments, something that was not performed in this study due to the invasive nature of the test. Another study limitation was that we did not collect stool samples for gut microbiota analyses.

The strength of this study was that it quantified the dietary composition of the patients in an objective manner. Using the FFQ, the investigators were able to compute the dietary pattern of the participants at baseline, and the analysis could easily be repeated at the end of the study, which allowed us to identify the reduction in total fat intake in the probiotics group. The use of serum-based LiverFAST also eliminated operator-dependent variability in assessing hepatic steatosis. We used multiple strains of *Lactobacillus and Bifidobacterium* at a high concentration in order to restore gut dysbiosis, while other studies used either single or multiple strains of a similar genus at a much lower concentration [10,29,30,31,32,33].

## 5. Conclusions

In this pilot study, the use of probiotics for a 6-month duration did not show any significant clinical improvement in NAFLD patients, namely hepatic steatosis, fibrosis, and activity scores, as well as biochemical blood tests. However, in the microenvironment of the small intestine, probiotics seemed to be able to stabilize the mucosal immune function, as shown by the reduced expression of CD8+ T lymphocytes in the placebo group, but not in the probiotics group. Additionally, probiotics were able to protect NAFLD patients against increased intestinal permeability, which was seen in the placebo group. Therefore, probiotics might play a complementary role in treating NAFLD. Further studies with larger sample sizes, a longer duration, and different probiotic strains are needed in order to evaluate the real benefit of probiotics in the management of NAFLD.

## Figures and Tables

**Table 1:** Contents of the probiotics used in the study.

Form	White Granules, Packed in a Sachet Form
Concentration	30 billion colony-forming units (CFU)
Strains	MCP^®^ BCMC^®^ strains consisting of *Lactobacillus acidophilus* BCMC 12,130 (107 mg), *Lactobacillus casei* subsp. BCMC 12,313 (107 mg), *Lactobacillus lactis* BCMC 12,451 (107 mg), *Bifidobacterium bifidum* BCMC 02290 (107 mg), *Bifidobacterium infantis* BCMC 02129 (107 mg) and *Bifidobacterium longum* BCMC 02120 (107 mg)
Product weight	3 g
Manufacturer	B-Crobes Laboratory Sdn. Bhd., GMP, manufactured in Malaysia

**Table 2:** Baseline characteristics of the studied patients. Values are presented as means (SD) for normally distributed data.

	Total (*n* = 39)	Probiotics (*n* = 17)	Placebo (*n* = 22)	*p*-Value
Age	53.44 (14.13)	54.70 (10.19)	52.47 (16.73)	0.63
Gender				
Male	28	11	17	
Female	11	6	5	
Diabetes mellitus				
Yes	19	9	10	
No	20	8	12	
Hypertension				
Yes	21	11	10	
No	18	6	12	
Metabolic characteristics				
Height, m	1.64 (0.08)	1.62 (0.09)	1.65 (0.07)	0.21
Weight, kg	76.70 (13.45)	75.00 (14.80)	78.03 (12.51)	0.49
BMI, kg/m^2^	29.62 (8.46)	31.33 (12.02)	28.30 (3.90)	0.63
Nutritional intake				
Average kcal	1731.82 (348.62)	1759.94 (408.39)	1710.09 (302.85)	0.66
Carbohydrate, g	202.79(40.86)	194.39 (46.82)	209.29 (35.35)	0.26
% of total kcal	46%	44%	48%	
Total fat, g	67.23 (20.78)	67.46 (21.81)	61.31 (19.84)	0.34
% of total kcal	33%	34%	32%	
Protein, g	85.16 (23.93)	91.64 (26.34)	80.16 (21.16)	0.14
% of total kcal	19%	20%	18%	
Serum biochemistry				
ALT, IU/L	72.02 (34.77)	70.29 (28.21)	73.36 (39.71)	0.78
AST, IU/L	46.92 (18.27)	44.35 (12.67)	48.90 (21.74)	0.44
GGT, IU/L	70.10 (54.41)	65.94 (34.07)	73.31 (66.69)	0.68
Triglycerides, mmol/L	2.06 (0.79)	2.04 (0.79)	2.09 (0.81)	0.84
Total Cholesterol, mmol/L	5.79 (0.89)	5.93(0.90)	5.68 (0.88)	0.38
Fasting glucose, mmol/L	5.34 (1.31)	5.13 (0.96)	5.50 (1.53)	0.38
Serum LiverFAST				
Steatosis score	0.64 (0.15)	0.67 (0.16)	0.62 (0.14)	0.41
Fibrosis score	0.30 (0.20)	0.28 (0.17)	0.33 (0.22)	0.46
Activity score	0.42 (0.23)	0.42 (0.21)	0.43 (0.24)	0.85
Transient elastography				
Liver stiffness, kPa	7.44 (2.76)	7.25 (2.76)	7.58 (2.82)	0.37
Controlled attenuated parameter, dB/m	333.51 (34.35)	339.11 (34.39)	329.18 (35.15)	0.71

ALT, alanine aminotransferase; AST, aspartate aminotransferase; BMI, body mass index; GGT, gamma-glutamyl transferase.

**Table 3:** Clinical parameters at baseline and the end of the study by intervention group.

	Probiotics (*n* = 17)			Placebo (*n* = 22)		
	Baseline	End of Study	*p*-Value	Baseline	End of Study	*p*-Value
Steatosis (CAP), dB/m	339.17 (33.58)	317.41 (40.37)	0.05	329.18 (35.15)	318.45 (45.37)	0.29
Liver stiffness, kPa	7.25 (2.76)	6.99 (2.74)	0.55	7.58 (2.82)	6.95 (2.19)	0.23
ALT, IU/L	70.29 (28.21)	84.29 (70.55)	0.26	73.36 (39.71)	74.50 (38.73)	0.84
AST, IU/L	44.35(12.67)	46.35 (23.19)	0.64	48.90 (21.74)	45.50 (25.80)	0.36
GGT, IU/L	65.94 (34.07)	72.17 (56.90)	0.45	73.31 (66.69)	74.63 (81.94)	0.81
* Steatosis score	0.72 (0.53–0.80)	0.76 (0.64–0.85)	0.06	0.67 (0.52–0.74)	0.73 (0.51–0.79)	0.053
* Fibrosis score	0.26 (0.15–0.40)	0.22 (0.18–0.36)	0.88	0.27 (0.15–0.45)	0.33 (0.15–0.36)	0.022
Activity score	0.42 (0.21)	0.41 (0.24)	0.78	0.43 (0.24)	0.44 (0.25)	0.57
* Body mass index, kg/m^2^	28.50 (25.0–31.60)	30.0 (26.20–32.90)	0.048	28.50 (25.05–31.15)	29.60 (25.85–32.03)	0.002
Triglycerides, mg/dL	2.04 (0.79)	1.94 (0.75)	0.55	2.09 (0.81)	2.01 (1.01)	0.66
Total cholesterol, mg/dL	5.93 (0.90)	6.17 (1.38)	0.31	5.68 (0.88)	5.74 (1.46)	0.79
Fasting glucose, mg/dL	5.13 (0.96)	5.6 (1.09)	0.06	5.50 (1.53)	5.14(0.68)	0.28

ALT, alanine aminotransferase; AST, aspartate aminotransferase; CAP, controlled attenuated parameter; GGT, gamma-glutamyl transferase. Values are presented as the mean (standard deviation), unless stated otherwise. * Values are presented as the median (interquartile range). Analysis was performed by Wilcoxon signed-rank tests due to a non-normal distribution.

**Table 4:** Mean changes in the study parameters between the groups from baseline to the end of the study.

	Probiotics (*n* = 17)		Placebo (*n* = 22)		
	Mean	SD	Mean	SD	*p*-Value
Steatosis, dB/m	−21.7	42.60	−10.72	46.64	0.45
Liver stiffness, kPa	−0.25	1.77	−0.62	2.37	0.59
ALT, IU/L	14.0	50.04	1.13	26.39	0.30
AST, IU/L	2.00	17.31	−3.40	17.09	0.33
GGT, IU/L	6.2	33	1.35	28.38	0.60
Steatosis score	0.049	0.09	0.042	0.11	0.83
Fibrosis score	0.01	0.10	0.06	0.09	0.18
Activity score	−0.12	0.15	0.015	0.13	0.56
Body mass index, kg/m^2^	0.7	1.46	0.82	1.06	0.81
Triglycerides, mg/dL	−0.10	0.68	−0.07	0.77	0.90
Total cholesterol, mg/dL	0.23	0.93	0.05	1.07	0.59
Fasting glucose, mg/dL	0.46	0.94	−0.44	1.31	0.03

ALT, alanine aminotransferase; AST, aspartate aminotransferase; GGT, gamma-glutamyl transferase. Values are presented as the mean (standard deviation). (−) denotes a reduction in the measurement at the end of the study compared with the baseline.

**Table 5:** Dietary intake from baseline to the end of the study within the groups.

	Probiotics (*n* = 17)			Placebo (*n* = 22)		
	Baseline	End of Study	*p*-Value	Baseline	End of Study	*p*-Value
Calories, kcal	1759.94 (408)	1645.03 (565)	0.39	1636.04 (457)	1637.11 (485)	0.98
Carbohydrates, g	194.39 (46.82)	215.31 (63.87)	0.26	209.29 (35.35)	213.24 (46.46)	0.70
Protein, g	91.64(26.34)	83.23 (23.77)	0.14	80.16 (21.16)	86.97 (21.75)	0.13
Total fat, g	67.46 (21.81)	56.82 (18.60)	<0.05	61.31 (19.84)	58.77 (16.96)	0.58

Value presented in Mean (standard deviation).

## Data Availability

The raw data of the clinical outcomes and IHC results are available from the corresponding author on reasonable request.
